# Implementation of a Prevention Bundle to Decrease Rates of *Staphylococcus aureus* Surgical Site Infection at 11 Veterans Affairs Hospitals

**DOI:** 10.1001/jamanetworkopen.2023.24516

**Published:** 2023-07-20

**Authors:** Hiroyuki Suzuki, Eli N. Perencevich, Stacey Hockett Sherlock, Gosia S. Clore, Amy M. J. O’Shea, Graeme N. Forrest, Christopher D. Pfeiffer, Nasia Safdar, Christopher Crnich, Kalpana Gupta, Judith Strymish, Gio Baracco Lira, Suzanne Bradley, Jose Cadena-Zuluaga, Michael Rubin, Marvin Bittner, Daniel Morgan, Aaron DeVries, Kelly Miell, Bruce Alexander, Marin L. Schweizer

**Affiliations:** 1 Center for Access and Delivery Research & Evaluation (CADRE), Iowa City Veterans’ Affairs Health Care System, Iowa City, Iowa; 2 Department of Internal Medicine, University of Iowa Carver College of Medicine, Iowa City; 3 Division of Infectious Disease, Rush University Medical Center, Chicago, Illinois; 4 Infectious Diseases Section, VA Portland Health Care System, Portland, Oregon; 5 Division of Infectious Diseases, OHSU, Portland, Oregon; 6 Division of Infectious Disease, Department of Medicine, University of Wisconsin School of Medicine and Public Health, Madison; 7 William S. Middleton Memorial Veterans Hospital, Madison, Wisconsin; 8 Division of Infectious Diseases, Department of Medicine, Boston VA Healthcare System, Boston, Massachusetts; 9 Center for Healthcare Organization and Implementation Research (CHOIR), Boston VA Healthcare System, Boston, Massachusetts; 10 Boston University School of Medicine, Boston, Massachusetts; 11 Department of Medicine, Harvard Medical School, Boston, Massachusetts; 12 Division of Infectious Diseases, Miller School of Medicine, University of Miami, Miami, Florida; 13 Hospital Epidemiology and Occupational Health Service, Miami VA Healthcare System, Miami, Florida; 14 Division of Infectious Diseases, Department of Internal Medicine, University of Michigan Medical School, Ann Arbor; 15 Infectious Diseases Section, Veterans Affairs Ann Arbor Healthcare System, Ann Arbor, Michigan; 16 South Texas Veterans Health Care System, San Antonio; 17 Long School of Medicine, UT Health San Antonio, San Antonio, Texas; 18 Department of Veterans’ Affairs, VA Salt Lake City Healthcare System, Salt Lake City, Utah; 19 Department of Internal Medicine, University of Utah School of Medicine, Salt Lake City; 20 Nebraska-Western Iowa Veterans Affairs Health Care System, Omaha, Nebraska; 21 Department of Medicine, Creighton University School of Medicine, Omaha, Nebraska; 22 Department of Epidemiology and Public Health, University of Maryland School of Medicine, Baltimore; 23 VA Maryland Health Care System, Baltimore; 24 Minneapolis VA Medical Center, Minneapolis, Minnesota

## Abstract

**Importance:**

While current evidence has demonstrated a surgical site infection (SSI) prevention bundle consisting of preoperative *Staphylococcus aureus* screening, nasal and skin decolonization, and use of appropriate perioperative antibiotic based on screening results can decrease rates of SSI caused by *S aureus*, it is well known that interventions may need to be modified to address facility-level factors.

**Objective:**

To assess the association between implementation of an SSI prevention bundle allowing for facility discretion regarding specific component interventions and *S aureus* deep incisional or organ space SSI rates.

**Design, Setting, and Participants:**

This quality improvement study was conducted among all patients who underwent coronary artery bypass grafting, cardiac valve replacement, or total joint arthroplasty (TJA) at 11 Veterans Administration hospitals. Implementation of the bundle was on a rolling basis with the earliest implementation occurring in April 2012 and the latest implementation occurring in July 2017. Data were collected from January 2007 to March 2018 and analyzed from October 2020 to June 2023.

**Interventions:**

Nasal screening for *S aureus*; nasal decolonization of *S aureus* carriers; chlorhexidine bathing; and appropriate perioperative antibiotic prophylaxis according to *S aureus* carrier status. Facility discretion regarding how to implement the bundle components was allowed.

**Main Outcomes and Measures:**

The primary outcome was deep incisional or organ space SSI caused by *S aureus*. Multivariable logistic regression with generalized estimating equation (GEE) and interrupted time-series (ITS) models were used to compare SSI rates between preintervention and postintervention periods.

**Results:**

Among 6696 cardiac surgical procedures and 16 309 TJAs, 95 *S aureus* deep incisional or organ space SSIs were detected (25 after cardiac operations and 70 after TJAs). While the GEE model suggested a significant association between the intervention and decreased SSI rates after TJAs (adjusted odds ratio, 0.55; 95% CI, 0.31-0.98), there was not a significant association when an ITS model was used (adjusted incidence rate ratio, 0.88; 95% CI, 0.32-2.39). No significant associations after cardiac operations were found.

**Conclusions and Relevance:**

Although this quality improvement study suggests an association between implementation of an SSI prevention bundle and decreased *S aureus* deep incisional or organ space SSI rates after TJAs, it was underpowered to see a significant difference when accounting for changes over time.

## Key Points

**Question:**

Is a surgical site infection (SSI) prevention bundle with facility-level discretion on its components associated with decreased *Staphylococcus aureus* deep incisional or organ space SSI after cardiac surgery or total joint arthroplasty?

**Findings:**

In this quality improvement study that implemented an SSI prevention bundle at 11 Veterans Affairs hospitals and included 23 005 surgical procedures, there was a significant association between the intervention and decreased deep incisional or organ space SSI rates among patients undergoing total joint arthroplasty but not among those undergoing cardiac surgery analyzed with a multivariable logistic regression model. The association was not observed when analyzed with an interrupted time-series model.

**Meaning:**

The findings of this study suggest that implementation of an SSI prevention bundle with facility-level discretion on its components may be associated with decreased deep incisional or organ space *S aureus* SSI after total joint arthroplasty, but further research is needed to investigate this association outside of randomized trial settings.

## Teaser

This quality improvement study assesses the association between implementation of a surgical site infection prevention bundle allowing for facility discretion regarding specific component interventions and *Staphylococcus aureus* deep incisional or organ space surgical site infection rates.

## Introduction

Surgical site infections (SSIs) are associated with significant morbidity and mortality, prolonged length of hospital stay, and readmission.^1^ *Staphylococcus aureus* is the most common etiology of adult SSIs, and specifically the most common pathogen causing SSIs among orthopedic (38.6%) and cardiac (27.0%) surgery patients.^2^ *S aureus* nasal carriage is an important risk factor for SSI after cardiac and orthopedic surgery.^3,4,5^ Studies have found that the majority of patients who develop SSIs with *S aureus* carry a genetically identical strain in their nares.^4,6^

A multicenter study involving 20 hospitals in the United States (STOP SSI study) found that implementation of an SSI prevention bundle decreased rates of deep incisional or organ space *S aureus* SSI by 42% among patients undergoing cardiac and orthopedic surgery.^7^ The bundle used in that study included chlorhexidine gluconate (CHG) bathing, *S aureus* nasal screening, nasal mupirocin decolonization for *S aureus* carriers, and perioperative antibiotic prophylaxis based on whether the patient was a methicillin-resistant *S aureus* (MRSA) carrier. However, it is well known that interventions are not one-size-fits-all and may need to be modified to address facility-level factors, such as laboratory capacity, and other barriers to implementation.^8^ Our prior research on this SSI prevention bundle found that barriers to bundle implementation could be overcome by adapting and tailoring strategies to stakeholders and settings.^9^ The aim of this quality improvement study was to implement an SSI prevention bundle while allowing for discretionary implementation of specific component interventions among 11 Veterans Affairs (VA) medical centers to assess the change in *S aureus* SSI rates when the prevention bundle was implemented outside a strict randomized clinical trial setting.

## Methods

### Ethics

This study was approved by the VA Central institutional review board as well as the Research and Development Committees of each participating hospital. It was preregistered on ClinicalTrials.gov(NCT02216227). This study was conducted using routinely collected data without direct patient contact and was deemed of minimal risk. A waiver of informed consent was obtained. We followed the SQUIRE Standards for Quality Improvement Reporting Excellence 2.0 (SQUIRE) guidelines for reporting.^10^

### Study Design and Patient Population

We conducted a quasi-experimental before-and-after study of all patients who underwent 1 of 3 primary surgical procedures: coronary artery bypass grafting (CABG), cardiac valve replacement, or total joint arthroplasty (TJA, which indicates total hip arthroplasty or total knee arthroplasty) at 11 VA medical centers. Before bundle implementation, SSI prevention efforts such as *S aureus* screening, CHG bathing, or perioperative antibiotic prophylaxis were not standardized.

### Intervention

The project held an in-person kickoff meeting to review project aims. Throughout the project, investigators and coordinators from all sites attended twice monthly conference calls to discuss local and national barriers and facilitators. All sites were given facilitation tools, such as information sheets, patient instructions, and flowcharts. Sites were guided through an implementation checklist to aid local implementation of the intervention. Barriers and facilitators of implementation were addressed when possible. Sites adapted based on shared lessons learned and peer coaching.

The recommended intervention included (1) preoperative nasal screening for *S aureus* within 30 days of the operation; (2) preoperative nasal decolonization of *S aureus* carriers with mupirocin twice daily for 5 days; (3) CHG bathing for 5 days prior to surgery for *S aureus* carriers and 2 days prior for noncarriers; (4) perioperative antibiotic prophylaxis with cefazolin unless the patient was known to be an MRSA carrier; and (5) perioperative antibiotic prophylaxis with both vancomycin and cefazolin for known MRSA carriers. However, sites were given flexibility in how to implement specific components of the bundled interventions (Table 1). For example, sites could choose to perform nasal screening for both MRSA and methicillin-susceptible *S aureus* (MSSA) or MRSA alone, since laboratory capacity for screening differed at each site. Sites used varying laboratory methods to determine MRSA and MSSA nasal colonization including standard culture method, culture using selective media for *S aureus* (CHROMagar), singleplex polymerase chain reaction (PCR), and multiplex PCR. Sites could choose between CHG wipes or liquid soap to use prior to surgery. Sites could also replace the 5-day regimen of mupirocin with a 1-time application of intranasal povidone-iodine. Sites were provided a table for recommended antibiotics for perioperative prophylaxis but were given flexibility in antibiotic choices (eTable 1 in Supplement 1).

**Table 1.** Components of Surgical Site Infections Prevention Bundle

Bundle component	Options	Frequency
Preoperative chlorhexidine bathing	Wipes or liquid body wash	Used for 5 d prior to surgery for *Staphylococcus aureus*–positive patients and night before and morning of surgery for *S aureus*–negative patients
*S aureus* nasal screening	MRSA only All *S aureus* No screening but universal decolonization	Within 30 d prior to surgery
Nasal decolonization	Mupirocin or povidone-iodine	Mupirocin: twice daily for 5 d prior to surgery Povidone-iodine: 1-time use prior to surgery
Perioperative prophylaxis	Provided a table for recommended antibiotics for perioperative prophylaxis but were given flexibility in the choice of antibiotics (eTable 1 in Supplement 1)	Perioperative

Abbreviation: MRSA, methicillin-resistant *Staphylococcus aureus*.

The intervention started when each site was prepared to begin. Thus, implementation of the intervention was on a rolling basis, with the earliest implementation occurring in April 2012 and the latest implementation occurring in July 2017.

### Data Collection

Data were collected on operations performed between January 1, 2010, and March 31, 2018, for cardiac operations, and between January 1, 2007, and March 31, 2018, for TJAs using *International Classification of Diseases, Ninth Revision *(*ICD-9*) and *International Statistical Classification of Diseases and Related Health Problems, Tenth Revision *(*ICD-10*) codes and *Current Procedural Terminology* codes (eTable 2 in Supplement 1). We excluded patients who had an established *S aureus* infection at the time of hospital admission for surgery. If a patient had more than 1 surgery during the study period, only the first surgery was included. Data from the VA’s integrated electronic medical records were pulled from the Corporate Data Warehouse through the VA Informatics and Computing Infrastructure.

### Outcomes

The primary outcome of interest was deep incisional or organ space SSI caused by *S aureus* within 90 days after index surgery. Organ space SSI included mediastinitis, endocarditis, and prosthetic joint infection. SSI was defined using the VA Surgical Quality Improvement Program (VASQIP) data collected monthly by experienced nurse data managers.^11^ VASQIP covers approximately 70% of operative cases, thus we assessed cases outside the VASQIP sample using a previously described fully automated SSI detection algorithm.^12^ That algorithm was also used to assess SSIs outside the 30-day follow-up period used by the VASQIP program.

### Statistical Analysis

Numbers of deep incisional or organ space *S aureus* SSI were summarized as frequencies and percentages for the preintervention period and the postintervention period. The χ^2^ test was used to compare the frequency of deep incisional or organ space *S aureus* SSI between the 2 periods. A patient-level multivariate generalized estimating equation (GEE) model with a logit link and robust SEs clustered within site was assessed. Odds ratios (ORs) and 95% CIs were reported. Similar models were applied to the outcome MRSA SSI.

Although we were not statistically powered to see a significant difference, we performed additional sensitivity analyses. First, we assessed the association between the intervention and deep incisional or organ space SSI among hospitals that screened for both MRSA and MSSA or hospitals that screened for only MRSA. Then an interrupted time-series (ITS) model was fit to account for baseline trends, autocorrelation, and time trends before and after the intervention implementation using a generalized linear model with a negative binomial distribution, log link function, and fixed site effects. Incidence rate ratio (IRR) and 95% CIs were reported.

All statistical tests were 2-sided, and statistical significance was defined as α < .05. Analyses were conducted using SAS version 9.4 software (SAS Institution).

## Results

In total, 23 005 surgical procedures (6696 cardiac operations and 16 309 TJAs) were included in this study (Table 2). All 11 hospitals implemented the intervention among patients who underwent TJA. Six hospitals implemented the intervention among patients who underwent cardiac surgery. Hospital D did not perform cardiac surgery but implemented *S aureus* testing and decolonization among patients sent to hospital E for cardiac surgery. The timing of implementation and specifics on how each hospital implemented the infection prevention bundle are summarized in Table 2. Six hospitals screened for all *S aureus* (MRSA and MSSA), and the other 5 hospitals screened for only MRSA. One hospital (hospital B) originally screened for MRSA only but stopped screening and decolonized all patients with povidone-iodine. Most of the hospitals used mupirocin for *S aureus* nasal decolonization, and 3 hospitals changed to povidone-iodine during the study period. Among all operations, 95 deep incisional or organ space *S aureus* SSIs were detected (0.41%). A total of 18 433 surgical procedures (80.1%) were screened by VASQIP nurse data managers, and 77 SSIs were detected (0.42%). In addition, 4572 surgical procedures (19.9%) were screened using the fully automated SSI detection algorithm, and 18 SSIs were detected (0.39%). There was no statistically significant difference in SSI detection rate when we compared VASQIP data with the detection algorithm (*P* = .82). Of 18 433 surgical procedures screened by VASQIP nurse data managers, 464 of 6489 (7.2%) cardiac operations and 65 of 11 940 (0.5%) TJAs were classified as urgent or emergent operations (*P* < .001).

**Table 2.** Hospital-Specific Information on Bundle Implementation

Hospital	Preoperative CHG bathing	*S aureus* nasal screening	Nasal decolonization	Bundle implementation start for cardiac surgery	Cardiac operations, No.	Bundle implementation start for TJA	TJAs, No.
A	CHG liquid body wash	MRSA (PCR) and MSSA (plate)	Mupirocin	Jan 2016	1246	Jan 2016	693
B	CHG liquid body wash	MRSA only (CHROMagar), eventually stopped screening	Mupirocin but changed to povidone-iodine after Sep 2016	NA	NA	Jan 2015	778
C	CHG liquid body wash	MRSA only (PCR for outpatients, spectra agar if screened as inpatient)	Mupirocin, but later changed to povidone iodine by mid-2015	Nov 2012	1720	Jul 2012	1851
D	CHG liquid body wash	MRSA and MSSA (PCR for both)	Mupirocin	Jul 2014	Included in site E numbers	Apr 2012	1388
E	CHG wipes	MRSA and MSSA (PCR for both)	Mupirocin	Apr 2017	470	Apr 2017	1121
F	CHG liquid body wash	MRSA (PCR), MSSA (CHROMagar)	Mupirocin	Jan 2015	968	Jan 2015	1171
G	CHG liquid body wash	MRSA only (PCR)	Mupirocin	NA	NA	Sep 2016	3725
H	CHG liquid body wash	MRSA only	Mupirocin	NA	NA	Apr 2016	1414
I	CHG liquid body wash	MRSA only (CHROMagar)	Mupirocin, but later changed to povidone iodine after Sep 2016	Apr 2015	1229	Apr 2015	1445
J	CHG liquid body wash	MRSA only (PCR)	Mupirocin	NA	NA	Apr 2015	1231
K	CHG liquid body wash	MRSA and MSSA (PCR for both)	Mupirocin	Jan 2015	1063	Jan 2015	1492

Abbreviations: CHG, chlorhexidine gluconate; MRSA, methicillin-resistant *Staphylococcus aureus*; MSSA, methicillin-susceptible *S aureus*; NA, not applicable; PCR, polymerase chain reaction; TJA, total joint arthroplasty.

There were 70 *S aureus* deep or organ space SSIs after the 15 212 cardiac and orthopedic operations in the preintervention period (0.46%); in contrast, there were 25 *S aureus* deep or organ space SSIs among 7793 cardiac and orthopedic operations in the intervention period (0.32%) (*P* = .12). In the GEE model adjusting for hospital-level correlation, we found there was not a statistically significant association between the intervention and the rate of *S aureus* deep or organ space SSIs (adjusted OR, 0.70; 95% CI, 0.46-1.06).

Among the 4255 cardiac operations performed in the preintervention period, 15 patients experienced *S aureus* mediastinitis or endocarditis (0.35%); in contrast, 10 patients experienced *S aureus* mediastinitis or endocarditis among the 2441 cardiac operations performed in the postintervention period (0.41%) (*P* = .71). Three hospitals reported a decreased proportion of SSIs, 2 hospitals reported an increased proportion of SSIs, and 1 hospital did not experience mediastinitis or endocarditis in either period (Figure). In the GEE model adjusting for hospital-level correlation, there was not a statistically significant association between the intervention and decreased mediastinitis or endocarditis among patients undergoing cardiac surgery (adjusted OR, 1.16; 95% CI, 0.70-1.92).

**Figure.**
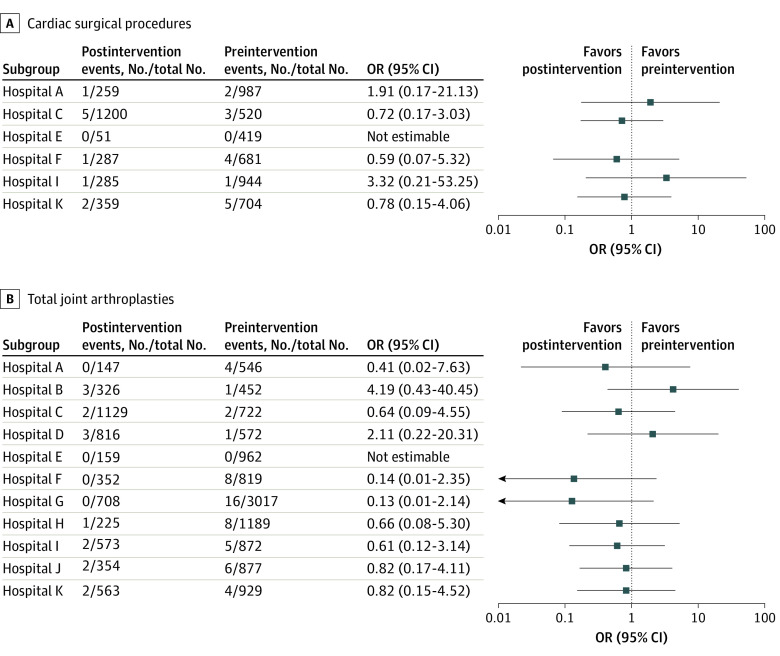
Number of Surgical Procedures and Surgical Site Infections During Preintervention and Postintervention Periods OR indicates odds ratio.

Among the 10 957 TJAs performed in the preintervention period, there were 55 deep or organ space *S aureus* SSIs (0.50%); among the 5352 TJAs performed in the postintervention period, there were 15 deep incisional or organ space *S aureus* SSIs (0.28%) (*P* = .04). Eight hospitals reported a decreased proportion of SSIs, 2 hospitals reported an increased proportion, and 1 hospital did not experience deep incisional or organ-space *S aureus* SSIs in either period (Figure). In the GEE model adjusting for hospital-level correlation, we found a statistically significant association between the intervention and decreased deep incisional or organ space *S aureus* SSI among patients undergoing TJA (adjusted OR, 0.55; 95% CI, 0.31-0.98). We performed a post hoc power analysis to determine the power necessary to reject the null hypothesis of no difference in deep incisional or organ space *S aureus* SSI, based on our results. We found that our analysis had 50% power to reject the null hypothesis of no difference.

We then evaluated the association of the intervention with deep incisional or organ space SSI caused by MRSA. Among patients undergoing cardiac surgery, there were 9 cases of MRSA mediastinitis or endocarditis in the preintervention period (0.21%) and 5 cases of MRSA mediastinitis or endocarditis in the postintervention period (0.20%) (adjusted OR, 0.73; 95% CI, 0.28-1.88). Among patients undergoing TJA, there were 24 deep incisional or organ space MRSA SSIs in the preintervention period (0.22%) and 6 deep incisional or organ space MRSA SSIs in the postintervention period (0.11%) (adjusted OR, 0.51; 95% CI, 0.19-1.41). These differences were not statistically significant.

### Sensitivity Analyses

We evaluated the difference in the association between the intervention and deep incisional or organ space *S aureus* SSI rates at hospitals that screened for both MRSA and MSSA nasal carriage and hospitals that screened for only MRSA. The associations were not statistically significant when assessing cardiac surgical procedures (screened for both MRSA and MSSA: OR, 1.05; 95% CI, 0.63-1.73; screened for MRSA: OR, 0.79; 95% CI, 0.46-1.34) nor TJAs (screened for both MRSA and MSSA: OR, 0.53; 95% CI, 0.17-1.69; screened for MRSA: OR, 0.60; 95% CI, 0.19-1.86).

The ITS model did not show a statistically significant associations between the intervention and deep incisional or organ space *S aureus* SSI among cardiac operations (adjusted IRR, 3.61; 95% CI, 0.73-18.00) or TJA (adjusted IRR, 0.88; 95% CI, 0.32-2.39) (eTables 3 and 4 in Supplement 1). In our post hoc power calculations, we found that the ITS model for patients undergoing TJA had 21% power to reject the null hypothesis of no difference.

## Discussion

This multicenter study evaluated the association between an SSI prevention bundle with facility-level discretion on its various components and *S aureus* deep incisional or organ space SSI rates. While we found a statistically significant difference among patients after TJA via GEE analysis, the association was not observed in the ITS analysis. In contrast, there was not a statistically significant association among patient after cardiac operations in either analysis.

Although both the GEE and ITS analyses were underpowered, post hoc power for the GEE and ITS models among patients after TJA were 50% and 21%, respectively. The lack of power in the ITS analysis was most likely derived from the rarity of the deep incisional or organ space SSI in each time point.

Our results corroborate the findings of the STOP SSI study, which evaluated a similar bundled intervention at 20 hospitals in Hospital Corporation of America–affiliated hospitals. The STOP SSI study used a well-defined bundle. Therefore, the focus was to show internal validity and to isolate the outcomes of the bundle from all external influences.^7,13,14^ In contrast, our study was an effectiveness study, in which we allowed much more flexibility in the choice of chlorhexidine bathing products, the method for screening of *S aureus*, and the use of mupirocin or povidone-iodine for decolonization. Our study not only assessed internal validity but also considered external validity in various clinical settings.^14^

In contrast to possible lower SSI rates after TJA, we did not observe changes in SSI rates after cardiac operations. This is in line with the STOP SSI study but discordant with a previous randomized clinical trial,^6^ which showed *S aureus* nasal screening and decolonization significantly decreased SSI rates after cardiac operations. The lack of an association among patients undergoing cardiac surgery is likely due to barriers to bundle adherence. While TJAs are usually performed as a nonemergent procedure, cardiac surgery, such as CABG surgery, often needs to be performed urgently. In our study, it was very difficult to implement the intervention among patients undergoing cardiac surgery, who unlike those undergoing orthopedic surgery, do not have routine preoperative clinic visits prior to surgery.

In the sensitivity analyses that evaluated the association between the intervention and SSI rates at hospitals that screened for both MRSA/MSSA nasal carriage and hospitals that screened for only MRSA, we found similar results although the associations were not statistically significant, with wide confidence intervals. This is likely due to the smaller number of outcomes in this subgroup. A previous meta-analysis suggested *S aureus* decolonization and bundled approaches were associated with lower rates of both MSSA SSI and MRSA SSI.^15^ The same meta-analysis found use of glycopeptides, such as vancomycin, as a perioperative antibiotic was protective against MRSA SSIs but not for MSSA SSIs. Several other studies reported that perioperative use of vancomycin was associated with higher rates of overall SSI compared with perioperative use of cefazolin.^16,17^ Based on the best existing data, a targeted bundled approach with *S aureus* screening, nasal decolonization, and use of the most appropriate perioperative antibiotic based on screening result (cefazolin for MSSA and vancomycin plus cefazolin for MRSA) may be the optimal strategy to decrease *S aureus* SSI, in combination with CHG bathing.

### Limitations

There are limitations in our study. First, we were unable to assess adherence because CHG was not always dispensed through pharmacy and because of variation in facility-level documentation of patient use of CHG and mupirocin at home. However, a survey at hospital D found that among TJA patients, 85% of patients were adherent to CHG bathing and 53% were adherent to mupirocin as directed.^18^ Similarly, we were unable to assess how thoroughly the intervention was implemented at each hospital. Qualitative interviews with health care workers at 3 of these hospitals found that facility-level adherence to the bundle varied due to barriers and facilitators, such as presence of a champion and level of buy-in among local staff.^9^ In addition, we did not have patient-level information to know whether the patients who developed an SSI received all appropriate components of the bundled intervention. All these limitations make it difficult to assess whether changes in SSI rates were attributable to the bundled intervention. Second, deep incisional or organ space *S aureus* SSIs are very rare events, so the study was underpowered to adjust for important confounding variables. This limitation undermines our ability to provide strong evidence for an association between the intervention and a decrease in deep incisional or organ space *S aureus* SSIs, even among patients undergoing TJA, because we were unable to statistically adjust for important confounders. The ITS analysis did not show a statistically significant difference for patients undergoing TJA although the incidence rate ratio was still less than 1. Third, we used the fully automated SSI detection algorithm for approximately 30% of cases outside the sample followed by VASQIP. Although the SSI detection algorithm was associated with moderate positive predictive values (52.5% for cardiac operations and 83.3% for TJAs) and high negative predictive values (99.8% for cardiac operations and 99.4% for TJAs), there was a possibility of ascertainment bias. However, the SSI rates were similar when assessed by VASQIP nurse managers and the automated SSI detection algorithm. Thus, we believe there is little concern for ascertainment bias. Additionally, our study was conducted within the Veterans Health Administration system where most patients were older males. This research was conducted at academically affiliated VA hospitals that were willing to implement this intervention and used the Veterans Health Administration systems’ existing infrastructure for MRSA surveillance and SSI tracking. It is unclear whether our findings are generalizable in other settings.

## Conclusions

Although we observed an association between implementation of an SSI prevention bundle with facility-level discretion on the various components and lower *S aureus* deep incisional or organ space SSI rates among patients after TJA, we were severely underpowered to show an association using the ITS model. More research should be done to investigate the association outside of randomized trial settings.

## Notes

Supplement 1. **eTable 1.** Decolonization and Perioperative Prophylaxis Instructions for Health Care Workers **eTable 2.** International Classification of Diseases (ICD)-9/10 Codes and Current Procedural Terminology (CPT) Codes Used to Identify Surgical Cases **eTable 3.** Interrupted Time-Series Analysis for Surgical Site Infections After Cardiac Operations **eTable 4.** Interrupted Time-Series Analysis for Surgical Site Infections After Total Joint Arthroplasties

Supplement 2.
Data Sharing Statement
