# Supplementary material for: Implementation of a Prevention Bundle to Decrease Rates of Staphylococcus aureus Surgical Site Infection at 11 Veterans Affairs Hospitals
Source: JAMA Netw Open. 2023 Jul 20;6(7):e2324516. doi: 10.1001/jamanetworkopen.2023.24516 (PMC10359960; doi:10.1001/jamanetworkopen.2023.24516)
Supplement: Supplement 1. — eTable 1. Decolonization and Perioperative Prophylaxis Instructions for Health Care Workers eTable 2. International Classification of Diseases (ICD)-9/10 Codes and Current Procedural Terminology (CPT) Codes Used to Identify Surgical Cases eTable 3. Interrupted Time-Series Analysis for Surgical Site Infections After Cardiac Operations eTable 4. Interrupted Time-Series Analysis for Surgical Site Infections After Total Joint Arthroplasties [file jamanetwopen-e2324516-s001.pdf]

Supplemental Online Content

Suzuki H, Perencevich EN, Hockett Sherlock S, et al. Implementation of a prevention bundle to decrease rates of *Staphylococcus aureus* surgical site infection at 11 Veterans Affairs hospitals. *JAMA Netw Open.* 2023;6(7):e2324516. doi:10.1001/jamanetworkopen.2023.24516

eTable 1. Decolonization and Perioperative Prophylaxis Instructions for Health Care Workers

eTable 2. International Classification of Diseases (ICD)-9/10 Codes and Current Procedural Terminology (CPT) Codes Used to Identify Surgical Cases

eTable 3. Interrupted Time-Series Analysis for Surgical Site Infections After Cardiac Operations

eTable 4. Interrupted Time-Series Analysis for Surgical Site Infections After Total Joint Arthroplasties

This supplemental material has been provided by the authors to give readers additional information about their work.

© 2023 Suzuki H et al. *JAMA Network Open.*

eTable 1. Decolonization and Perioperative Prophylaxis Instructions for Health Care Workers

Decolonization of carriers is defined as 5 days of intra-nasal mupirocin 2% ointment administered twice daily according to the manufacturer's instructions. The patient or healthcare worker will apply the ointment to the interior of each nostril. If nasal devices are in place (e.g., nasal intubation, naso-gastric tubes) the mupirocin will be applied around the tubing and the nostril will be gently massaged to distribute the ointment. Outpatient CHG bathing, is an adjunctive approach to decolonization and will be recommended to the patient and instructions will be given. The patient carrying MRSA (and potentially MSSA) will be asked to bathe using 4% CHG solution on each of 5 days prior to the surgery date. If the surgery is emergent, the CHG bathing will occur in the inpatient setting before the surgical procedure and will be done both the evening before and the morning of the surgery if possible. If the patient has been hospitalized for up to 5 days prior to the surgery or if the surgery was a scheduled procedure, CHG bathing will be done for the 5 days prior to the surgery date.

Dosing and redosing recommendations are based on the updated American Society of Health System Pharmacists (ASHP) 2013 guidelines. Antimicrobial dosing will be weight-based and redosing guidelines are based on the drug's half-life, that is, the algorithm recommends giving a second dose when the operation's duration is greater than 2-times the drug's half-life.

Antimicrobial	Adult dose	Drug half-life in adults with normal renal function (hours)	Timing of the first dose of antimicrobial therapy	Infusion duration	Redosing interval (from initiation of the preoperative dose in hours)	Duration of Prophylaxis
Cefazolin	2 g<br>3g for ≥ 120 kg	1.2-2.2	≤60 min. before incision	3-5 min (IV push)<br>15-60 min (IV infusion)	4	Less than 24 hours after end of surgery

© 2023 Suzuki H et al. *JAMA Network Open.*

Cefuroxime *(may substitute for cefazolin)*	1.5g IV	1-2	≤60 min. before incision	3-5 min (IV push)<br><br>15-60 min (IV infusion)	4	Less than 24 hours after end of surgery
Vancomycin	15 mg/kg	4-8	≤120 min. before incision	1 g over 60 min (use longer infusion time if dose >1 g)	12	No post-op doses

Reference: Bratzler DW, Dellinger EP, Olsen KM, et al. Clinical practice guidelines for antimicrobial prophylaxis in surgery. American Journal of Health-System Pharmacy. Feb 1 2013;70(3):195-283.

Allergy Information:

- May substitute cefurozime for cefazolin; uncomfirmed beta-lactam allergy does not preclude the use of cefazolin. For a confirmed beta-lactam allergy, use vancomycin 15mg/kg (<120 minutes before operation) in place of cefazolin and add either gentamicin 5mg/kg, or aztreonam 2Gm <60 minutes before operation/incision.
- For vancomycin allergy, may use daptomycin (4mg/kg) in combination with cefazolin (if not beta-lactam allergic) for preoperative prophylaxis <60 minutes before operation/incision. If also beta-lactam allergic, use gentamicin 5mg/kg, or aztreonam 2Gm <60 minutes before operation/incision in combination with the daptomycin. Vancomycin, daptomycin, or gentamicin prophylaxis should not be continued after the operation. Cefazolin and aztreonam should be discontinued within 24-hrs of the operation.
- Discontinue if patient experiences any side effects or allergic reaction to mupirocin or chlorhexidine gluconate.

© 2023 Suzuki H et al. *JAMA Network Open.*

eTable 2. *International Classification of Diseases* (*ICD*)-*9/10* Codes and *Current Procedural Terminology* (*CPT*) Codes Used to Identify Surgical Cases

	ICD-9 codes	ICD-10 codes	CPT codes
Cardiac surgeries	35.11, 35.12, 35.13, 35.14, 35.21, 35.22, 35.23, 35.24, 35.25, 35.26, 35.27, 35.28, 35.31, 35.32, 35.33, 35.51, 35.53, 35.61, 35.62, 35.72, 35.99, 36.11, 36.12, 36.13, 36.14, 36.15, 36.16, 36.17, 36.19	025D0ZZ, 027F04Z, 027F0DZ, 027F0ZZ, 027G04Z, 027G0DZ, 027G0ZZ, 027H04Z, 027H0DZ, 027H0ZZ, 027J04Z, 027J0DZ, 027J0ZZ, 02890ZZ, 028D0ZZ, 02BD0ZZ, 02CD0ZZ, 02CF0ZZ, 02CG0ZZ, 02CH0ZZ, 02CJ0ZZ, 02N90ZZ, 02ND0ZZ, 02NF0ZZ, 02NG0ZZ, 02NH0ZZ, 02NJ0ZZ, 02Q90ZZ, 02QD0ZZ, 02QF0ZZ, 02QG0ZZ, 02QH0ZZ, 02QJ0ZZ 02QM0ZZ, 02R907Z, 02R908Z, 02R90JZ, 02R90KZ, 02RD07Z, 02RD08Z, 02RD0JZ, 02RD0KZ, 02RF07Z, 02RF08Z, 02RF0JZ, 02RF0KZ, 02RG07Z, 02RG08Z, 02RG0JZ, 02RG0KZ, 02RH07Z, 02RH08Z, 02RH0JZ, 02RH0KZ, 02RJ07Z, 02RJ08Z, 02RJ0JZ, 02RJ0KZ, 02RM07Z, 02RM0JZ, 02RM0KZ, 02T90ZZ, 02TD0ZZ, 02TH0ZZ, 02U507Z, 02U508Z, 02U50JZ, 02U50KZ, 02U907Z, 02U908Z, 02U90JZ, 02U90KZ, 02UD07Z, 02UD08Z, 02UD0JZ, 02UD0KZ, 02UF07Z, 02UF08Z, 02UF0JZ, 02UF0KZ, 02UG07Z, 02UG08Z, 02UG0JZ, 02UG0KZ, 02UH07Z, 02UH08Z, 02UH0JZ, 02UH0KZ, 02UJ07Z, 02UJ08Z, 02UJ0JZ, 02UJ0KZ, 02UM07Z, 02UM0JZ, 02UM0KZ,	0259T, 27130, 27132, 27445, 27447, 33030, 33031, 33250, 33251, 33256, 33260, 33261, 33300, 33305, 33310, 33315, 33320, 33321, 33322, 33330, 33332, 33335, 33350, 33400, 33401, 33403, 33405, 33406, 33410, 33411, 33412, 33413, 33414, 33417, 33420, 33422, 33425, 33426, 33427, 33430, 33450, 33452, 33460, 33463, 33464, 33465, 33468, 33476, 33478, 33496, 33500, 33501, 33503, 33504, 33510, 33511, 33512, 33513, 33514, 33516, 33517, 33518, 33519, 33521, 33522, 33523, 33530, 33533, 33534, 33535, 33536, 33572, 33600, 33602, 33640, 33641, 33643, 33660, 33665, 33681, 33682, 33684, 33688, 33692, 33694, 33696, 33697, 33698, 33702, 33710, 33720, 33722, 33735, 33736, 33737, 33738, 33739, 33813, 33814, 33852 33853, 33855, 33860, 33861, 33863, 33864, 33870, 33875, 33877, 33891, 33922, 33926, 33999, 34502, 35180, 35182, 35184, 35188, 35189, 35190, 35201, 35206, 35207, 35211, 35216, 35221, 35226, 35231, 35236, 35241, 35246, 35251,

© 2023 Suzuki H et al. *JAMA Network Open.*

		021009C, 02009F, 021009W, 02100A3, 02100A8, 02100A9, 02100AC, 02100AF, 02100AW, 02100J3, 02100J8, 02100J9, 02100JC, 02100JF, 02100JW, 02100K3, 02100K8, 02100K9, 02100KC, 02100KF, 02100KW, 02100Z3, 02100Z8, 02100Z9, 02100ZC, 02100ZF, 021109C, 021109F, 021109W, 02110A3, 02110A8, 02110A9, 02110AC, 02110AF, 02110AW, 02110J3, 02110J8, 02110J9, 02110JC, 02110JF, 02110JW, 02110K3, 02110K8, 02110K9, 02110KC, 02110KF, 02110KW, 02110Z3, 02110Z8, 02110Z9, 02110ZC, 02110ZF, 021209C, 021209F, 021209W, 02120A3, 02120A8, 02120A9, 02120AC, 02120AF, 02120AW, 02120J3, 02120J8, 02120J9, 02120JC, 02120JF, 02120JW, 02120K3, 02120K8, 02120K9, 02120KC, 02120KF, 02120KW, 02120Z3, 02120Z8, 02120Z9, 02120ZC, 02120ZF, 021309C, 021309F, 021309W, 02130A3, 02130A8, 02130A9, 02130AC, 02130AF, 02130AW, 02130J3, 02130J8, 02130J9, 02130JC, 02130JF, 02130JW, 02130K3, 02130K8, 02130K9, 02130KC, 02130KF, 02130KW, 02130Z3, 02130Z8, 02130Z9,	35256, 35261, 35266, 35271, 35276, 35281, 35286, 35506, 35508, 35518, 35523, 35526, 35533, 35539, 35600, 35626, 35650, 35685, 4110F, 92987, 92992, 92993, 93650, 93651, 93652, 93799, S2205, S2206, S2207, S2208, S2209

© 2023 Suzuki H et al. *JAMA Network Open.*

		02130ZC, 02130ZF, 210093, 210098, 210099, 211093, 211098, 211099, 212093, 212098, 212099, 213093, 213098, 213099	
Total Joint Arthroplasty	00.74, 00.75, 00.76, 00.77, 00.85, 00.86, 00.87, 81.51, 81.52, 81.54	0SR9019, 0SR901A, 0SR901Z, 0SR9029, 0SR902A, 0SR902Z, 0SR9039, 0SR903A, 0SR903Z, 0SR9049, 0SR904A, 0SR904Z, 0SR907Z, 0SR90J9, 0SR90JA, 0SR90JZ, 0SR90KZ, 0SRA009, 0SRA00A, 0SRA00Z, 0SRA019, 0SRA01A, 0SRA01Z, 0SRA039, 0SRA03A, 0SRA03Z, 0SRA07Z, 0SRA0J9, 0SRA0JA, 0SRA0JZ, 0SRA0KZ, 0SRB019, 0SRB01A, 0SRB01Z, 0SRB029, 0SRB02A, 0SRB02Z, 0SRB039, 0SRB03A, 0SRB03Z, 0SRB049, 0SRB04A, 0SRB04Z, 0SRB07Z, 0SRB0J9, 0SRB0JA, 0SRB0JZ, 0SRB0KZ, 0SRC07Z, 0SRC0J9, 0SRC0JA, 0SRC0JZ, 0SRC0KZ, 0SRD07Z, 0SRD0J9, 0SRD0JA, 0SRD0JZ, 0SRD0KZ, 0SRE009, 0SRE00A, 0SRE00Z, 0SRE019, 0SRE01A, 0SRE01Z, 0SRE039, 0SRE03A, 0SRE03Z, 0SRE07Z, 0SRE0J9, 0SRE0JA, 0SRE0JZ, 0SRE0KZ, 0SRR019, 0SRR01A, 0SRR01Z, 0SRR039, 0SRR03A, 0SRR03Z, 0SRR07Z, 0SRR0J9, 0SRR0JA, 0SRR0JZ, 0SRR0KZ, 0SRS019, 0SRS01A, 0SRS01Z, 0SRS039, 0SRS03A, 0SRS03Z, 0SRS07Z, 0SRS0J9, 0SRS0JA, 0SRS0JZ, 0SRS0KZ, 0SRT07Z, 0SRT0J9,	27130, 27132, 27445, 27447

		0SRT0JA, 0SRT0JZ, 0SRT0KZ, 0SRU07Z, 0SRU0J9, 0SRU0JA, 0SRU0JZ, 0SRU0KZ, 0SRV07Z, 0SRV0J9, 0SRV0JA, 0SRV0JZ, 0SRV0KZ, 0SRW07Z, 0SRW0J9, 0SRW0JA, 0SRW0JZ, 0SRW0KZ, 0SU90BZ, 0SUA0BZ, 0SUB0BZ, 0SUE0BZ, 0SUR0BZ, 0SUS0BZ	

© 2023 Suzuki H et al. *JAMA Network Open.*

eTable 3. Interrupted Time-Series Analysis for Surgical Site Infections After Cardiac Operations

**Adjusted Model**	**Coefficient**	**Incident rate ratio (95% CI)**	**p-value**
Intercept	-1.904	0.14 (0.05-0.45)	<0.001
Time	-0.025	0.98 (0.95-1.00)	0.10
Intervention (Ref: Pre-intervention)	1.284	3.61 (0.73-18.00)	0.12
Post-Intervention Time	-0.018	0.98 (0.93-1.04)	0.54
Time + Post-Intervention Time	-0.042	0.96 (0.91-1.01)	0.11
Hospitals (Ref: Hospital K)			
Hospital A	-0.787	0.46 (0.11-1.89)	0.28
Hospital C	-0.020	0.98 (0.28-3.38)	0.97
Hospital F	-0.307	0.74 (0.22-2.49)	0.62
Hospital I	-1.269	0.28 (0.06-1.43)	0.13

* Hospital E was removed from this analysis with no surgical site infections in both pre-intervention and intervention periods

© 2023 Suzuki H et al. *JAMA Network Open.*

eTable 4. Interrupted Time-Series Analysis for Surgical Site Infections After Total Joint Arthroplasties

**Adjusted Model**	**Coefficient**	**Incident Rate Ratio (95% CI)**	**p-value**
Intercept	-3.18	0.04 (0.02-0.11)	<0.001
Time	0.003	1.00 (0.99-1.01)	0.57
Intervention (Ref: Pre-intervention)	-0.133	0.88 (0.32-2.39)	0.79
Post-Intervention Time	-0.015	0.99 (0.95-1.02)	0.44
Time + Post-Intervention Time	-0.012	0.99 (0.95-1.03)	0.51
Hospitals (Ref: Hospital K)			
Hospital A	-0.456	0.63 (0.18-2.29)	0.49
Hospital B	-0.403	0.67 (0.19-2.41)	0.54
Hospital C	-0.221	0.80 (0.22-2.98)	0.74
Hospital D	-0.198	0.82 (0.22-3.08)	0.77
Hospital F	0.286	1.33 (0.45-3.91)	0.60
Hospital G	0.902	2.46 (0.93-6.51)	0.07
Hospital H	0.343	1.41 (0.49-4.06)	0.53
Hospital I	0.140	1.15 (0.30-3.50)	0.80
Hospital J	0.275	1.32 (0.45-3.88)	0.62

* Hospital E was removed from this analysis with no surgical site infections in both pre-intervention and intervention periods

© 2023 Suzuki H et al. *JAMA Network Open.*
