# Supplementary material for: Implementation of a Prevention Bundle to Decrease Rates of Staphylococcus aureus Surgical Site Infection at 11 Veterans Affairs Hospitals
Source: JAMA Netw Open. 2023 Jul 20;6(7):e2324516. doi: 10.1001/jamanetworkopen.2023.24516 (PMC10359960; doi:10.1001/jamanetworkopen.2023.24516)
Supplement: Supplement 2. — Data Sharing Statement [file jamanetwopen-e2324516-s002.pdf]

Data Sharing Statement

Suzuki. Implementation of a Prevention Bundle to Decrease Rates of Staphylococcus aureus Surgical Site Infection at 11 Veterans Affairs Hospitals. *JAMA Netw Open*. Published July 20, 2023. doi:10.1001/jamanetworkopen.2023.24516

Data
Data available: No

Additional Information
Explanation for why data not available: All data reside behind a secure VA server and cannot be removed from that server.
